# Gestational diabetes mellitus and its associated factors among women of advanced maternal age in Malaysia: Findings from a national survey

**DOI:** 10.1371/journal.pone.0333005

**Published:** 2025-09-22

**Authors:** Chean Tat Chong, Lalitha Palaniveloo, Sulhariza Husni Zain, Muhamad Khairul Nazrin Khalil, Kishwen Kanna Yoga Ratnam

**Affiliations:** 1 Centre for Nutrition Epidemiology Research, Institute for Public Health, National Institutes of Health, Ministry of Health, Setia Alam, Malaysia; 2 Centre for Family Health Research, Institute for Public Health, National Institutes of Health, Ministry of Health, Setia Alam, Malaysia; 3 Centre for Non-Communicable Disease Research, Institute for Public Health, National Institutes of Health, Ministry of Health, Setia Alam, Malaysia; Universiti Sains Malaysia, MALAYSIA

## Abstract

Gestational diabetes mellitus (GDM) is a growing public health concern, particularly among women with advanced maternal age. Understanding the prevalence and associated sociodemographic factors is crucial for targeted interventions. This study aimed to determine the prevalence of GDM and its association with sociodemographic factors among Malaysian women with advanced maternal age. This study utilized data from the National Health and Morbidity Survey 2022: Maternal and Child Health, a nationwide cross-sectional survey employing a two-stage stratified cluster sampling design. GDM was identified based on the result of a modified oral glucose tolerance test (MOGTT) recorded in the mother’s antenatal book. The 75-g MOGTT was performed according to the Clinical Practice Guidelines for the Management of Diabetes in Pregnancy in Malaysia. Sociodemographic variables, including ethnicity, locality, education, employment, and household income, were analysed. Multiple logistic regression was performed to identify factors associated with GDM. The prevalence of GDM among women with advanced maternal age in Malaysia was 33.7% (95% CI: 30.9%−36.5%). Ethnicity was significantly associated with GDM, with Indian women showing the highest prevalence (48.8%) and odds (AOR: 7.31, 95% CI: 2.58–20.72; P < 0.001). Working status was another significant factor, with non-working women having higher odds of GDM compared to working women (AOR: 1.34, 95% CI: 1.01–1.77; P = 0.003). No significant associations were observed for locality, educational level, or household income. The high prevalence of GDM among women with advanced maternal age in Malaysia underscores the urgent need for targeted interventions, particularly among high-risk ethnic groups. Public health strategies should prioritize early screening, culturally tailored programs, and community-based initiatives to address this growing burden. Future research should explore behavioural and genetic determinants to further inform policy and practice.

## Introduction

Gestational Diabetes Mellitus (GDM) is a growing public health concern especially among women with advanced maternal age (AMA) [1,2], typically defined as pregnancy occurring at 35 years or older, is a well-established risk factor for GDM [3–5]. GDM, characterized by glucose intolerance first identified during pregnancy, poses significant risks to both maternal and neonatal health. Women diagnosed with GDM are at increased risk of developing type 2 diabetes later in life, and their children may face long-term health challenges, including obesity and metabolic disorders [2,6,7].

Women of AMA face increased physiological challenges during pregnancy, including reduced insulin sensitivity and a higher likelihood of pre-existing metabolic conditions, which contribute to GDM development [3–5]. Studies have consistently shown that the prevalence of GDM rises with maternal age, likely due to the cumulative effect of aging on pancreatic beta-cell function and the body’s ability to regulate glucose levels. Additionally, pregnancies at advanced ages are often associated with other complications, such as hypertension and obesity, further compounding the risk.

A globally standardized diagnostic protocol for GDM has yet to be universally accepted, making international comparisons challenging [2,8]. In 2010, the International Association of Diabetes in Pregnancy Study Groups (IADPSG) introduced criteria, which the World Health Organization (WHO) endorsed in 2013, to differentiate between two categories of women diagnosed with hyperglycaemia during pregnancy [2]. One category includes women who meet the diagnostic criteria for diabetes outside of pregnancy, referred to as having “overt diabetes” by the IADPSG and “diabetes in pregnancy” by the WHO.

GDM is driven by multiple factors, including the rising rates of obesity, sedentary lifestyles, and changing dietary patterns [9]. The country’s ethnically diverse population—comprising Malays, Chinese, Indians, and other ethnicity groups—presents unique sociodemographic variables that influence GDM prevalence. Studies suggest that age, body mass index (BMI), and family history of diabetes play a critical role, but the impact of socioeconomic factors such as education level, income, and access to healthcare requires further investigation [10].

In Malaysia, the prevalence of GDM increased from 12.5% in 2016 to 27.1% in 2022 [11,12]. According to the Clinical Practice Guidelines for the Management of Diabetes in Pregnancy in Malaysia, GDM is diagnosed using a 75-gram modified oral glucose tolerance test (MOGTT) [13]. This test is administered at the initial booking visit, and if the result is negative, it is repeated between 24 and 28 weeks of gestation. Understanding the prevalence of GDM and its sociodemographic determinants among women with advanced maternal age is essential to guide healthcare policies and interventions aimed at reducing maternal and neonatal complications. This study aims to explore the prevalence of GDM among women in Malaysia with advanced maternal age and examine the sociodemographic factors associated with this condition, providing valuable insights into the management and prevention strategies for GDM in Malaysia.

## Materials and methods

### Study design

This study obtained permission on December 2024 to perform secondary data analysis on the National Health and Morbidity Survey 2022: Maternal and Child Health (NHMS 2022: MCH). This cross-sectional nationwide survey was conducted between August to October 2022 and it employed a two-stage stratified cluster sampling design covering all states and federal territories in Malaysia to ensure national representativeness. The primary stratum consisted of states and federal territories, while the secondary stratum differentiated between urban and rural areas. Enumeration Blocks (EBs) were used as the primary sampling units (PSUs), with living quarters (LQs) within each selected EB serving as the secondary sampling units (SSUs). The Department of Statistics Malaysia (DOSM) randomly selected the PSUs based on the required sample size. The survey involved a total of 25,413 respondents, with an overall response rate of 74.9%. A total of 55 data collection teams were deployed, each consisting of a field supervisor, trained nurses, and research assistants. Prior to data collection, all team members underwent standardized training to ensure data quality and consistency. The full methodology for the NHMS 2022: MCH is detailed in the report [14].

### Questionnaire and survey instruments

The survey utilized structured, validated questionnaires administered through face-to-face interviews via mobile devices, as well as a self-administered questionnaire (SAQ). Hardcopy versions of the questionnaires were prepared as backups in case of technical issues. The questionnaires were pre-tested in both Malay and English, and a manual outlining the flow of the questionnaire and definitions of key terms was provided to guide the data collectors.

### Variable definition

Gestational diabetes mellitus (GDM) was identified based on the result of a modified oral glucose tolerance test (MOGTT) recorded in the mother’s antenatal book. GDM is diagnosed using a 75g modified oral glucose tolerance test (MOGTT) at booking where fasting plasma glucose (FPG) levels are ≥ 5.1 mmol/L and/or a 2-hour postprandial (2-HPP) glucose level is ≥ 7.8 mmol/L [15]. A repeat test conducted at 24–28 weeks of gestation if the initial result is negative. This is aligned with the International Association of Diabetes and Pregnancy Study Groups (IADPSG) diagnostic criteria for GDM [16] but only FBG and 2-HPP glucose were taken.

The sociodemographic data were categorized as follows: Locality was classified into two categories: urban and rural. Ethnicity was grouped into Malay, Chinese, Indian, Other Bumiputeras (inclusive of *Orang Asli* from peninsular Malaysia, Bumiputera Sabah and Bumiputera Sarawak), and others. Marital status was categorized into two groups: single, separated, divorced, or widowed; and married or cohabitating. Educational level was divided into four categories: no formal education, primary education, secondary education, and tertiary education. Employment status was classified into two groups: employed and unemployed (inclusive of housewife, student or not working). Household income was categorized into three groups based on the Department of Statistics Malaysia (DOSM) classification: Bottom 40% (B40) representing low income, Middle 40% (M40) representing middle income, and Top 20% representing high income (T20) [17].

### Statistical analysis

Statistical analysis was conducted using IBM SPSS Statistics (version 28, Chicago, Illinois, USA). Out of a total of 6,146 female respondents, only 1,754 were women of advanced maternal age and were included in the present analysis. A complex sampling analysis was employed, incorporating individual weights to account for unequal probabilities of selection (design weights), non-response adjustments, and post-stratification calibration using the 2022 population projections provided by the Department of Statistics Malaysia (DOSM). Descriptive statistics were employed to summarize the data. The Pearson’s χ² test was used to assess differences between GDM and sociodemographic variables. To identify factors associated with GDM, a multiple logistic regression model was applied, adjusting for potential confounders. Variables with a P-value less than.25 were included in the final model, and adjusted odds ratios (AORs) were calculated for each variable. A P-value of less than.25 was used to account for residual confounding. Both crude odds ratios (CORs) and AORs were reported with 95% confidence intervals (CIs), and P-values less than.05 were considered statistically significant.

### Ethical approval

The Medical Research and Ethics Committee of the Ministry of Health Malaysia approved the methodology, protocol, and procedures for the NHMS 2022: MCH. The survey was registered with the National Medical Research Registry under NMRR-20-959-53329. Written informed consent was obtained from all participants prior to their interviews during the NHMS 2022: MCH data collection.

## Results

A total of 1754 respondents were included in this study. The prevalence of GDM among women with advanced maternal age in Malaysia is presented in Table 1. Overall, the prevalence of gestational diabetes mellitus (GDM) among women with advanced maternal age in Malaysia was 33.7% (95% CI: 30.9%−36.5%). Analysis of demographic and socioeconomic factors revealed notable variations. There was no significant difference in GDM prevalence between urban (34.4%) and rural areas (31.8%) (P = 0.390). However, ethnicity was significantly associated with GDM prevalence (P < 0.001). Indian women had the highest prevalence (48.8%), followed by Malay (35.2%), Chinese (29.3%), Other Bumiputera (28.1%), and other ethnicities (12.7%). For marital status, married or cohabiting women having a higher prevalence (33.9%) compared to single, separated, divorced, or widowed women (16.7%) (P = 0.043). In contrast, educational level, working status, and household income were not significantly associated with GDM prevalence. The prevalence was comparable across education levels: no formal education (22.8%), primary (29.2%), secondary (34.5%), and tertiary (34.0%) (P = 0.568). Similarly, non-working women (36.0%) and working women (31.4%) had no significant difference in prevalence (P = 0.090). Household income groups also showed similar prevalence rates: B40 (33.2%), M40 (35.6%), and T20 (32.8%) (P = 0.747).

**Table 1:** Prevalence of GDM among women with AMA by sociodemographic characteristics in Malaysia, *n* = 1,754.

Characteristics	Unweighted Count, *n*	Estimated population	Prevalence (%)	95% CI (%)	P value
Overall	611	73659	33.7	30.9-36.5	
Locality Urban Rural	440 171	53797 19862	34.4 31.8	31.1-37.9 27.0-36.9	0.390
Ethnicity Malay Chinese Indian Other Bumiputera Other	510 18 28 44 11	57994 3424 4594 6294 1353	35.2 29.3 48.8 28.1 12.7	32.1-38.4 17.8-44.2 35.2-62.6 21.3-36.0 6.7-22.9	<0.001
Marital Status Single/Separated/ Divorcee/Widow Married/Cohabiting	6 605	515 73144	16.7 33.9	7.3-33.9 31.1-36.8	0.043
Educational Level No formal education Primary Secondary Tertiary	7 25 294 274	754 3791 34694 33165	22.8 29.2 34.5 34.0	10.4-42.9 20.0-40.4 30.5-38.7 30.0-38.2	0.568
Working Status Not working Working	346 255	40791 31724	36.0 31.4	32.3-39.9 27.5-35.5	0.090
Household Income B40 M40 T20	433 134 43	51181 17744 4615	33.2 35.6 32.8	30.2-36.5 29.4-42.4 24.1-42.8	0.747

In Table 2, multivariate analysis revealed the factors associated with GDM among women with advanced maternal age in Malaysia. The factors significantly associated included ethnicity and working status. Indian women had the highest odds of developing GDM (AOR: 7.31, 95% CI: 2.58–20.72; P < 0.001), followed by Malay (AOR: 4.42, 95% CI: 1.84–10.65; P < 0.001), Chinese (AOR: 3.56, 95% CI: 1.18–10.75; P = 0.024), and Other Bumiputera (AOR: 3.01, 95% CI: 1.24–7.32; P = 0.015), compared to the other ethnicities. Additionally, non-working women had significantly higher odds of developing GDM compared to working women (AOR: 1.34, 95% CI: 1.01–1.77; P = 0.003). In contrast, other factors were not significantly associated with GDM. Locality (COR: 1.13, 95% CI: 0.86–1.48; P = 0.390) and marital status (AOR: 2.21, 95% CI: 0.88–5.51; P = 0.090) showed no significant differences. Educational level also did not have a significant association, with no formal education (AOR: 1.24, 95% CI: 0.45–3.42; P = 0.681), primary (AOR: 0.87, 95% CI: 0.51–1.49; P = 0.602), and secondary (AOR: 0.96, 95% CI: 0.72–1.28; P = 0.796) levels showing similar odds. Similarly, household income showed no significant association across income categories, with B40 (COR: 1.02, 95% CI: 0.65–1.59; P = 0.928) and M40 (COR: 1.13, 95% CI: 0.69–1.86; P = 0.619) being comparable to the T20 group.

**Table 2:** Factors associated with GDM among women with advanced maternal age in Malaysia.

Characteristics	COR (95% CI)	P value	AOR (95% CI)	P value
Locality Urban Rural	1.13 (0.86, 1.48) Ref	0.390		
Ethnicity Malay Chinese Indian Other Bumiputera Other	3.73 (1.81, 7.68) 2.84 (1.07, 7.48) 6.55 (2.56, 16.72) 2.68 (1.24, 5.78) Ref	<0.001 0.035 <0.001 0.012	4.42 (1.84, 10.65) 3.56 (1.18, 10.75) 7.31 (2.58, 20.72) 3.01 (1.24, 7.32) Ref	<0.001 0.024 <0.001 0.015
Marital Status Single/Separated/Divorcee/Widow Married/Cohabiting	Ref 2.56 (0.99, 6.55)	0.051	Ref 2.21 (0.88, 5.51)	0.090
Educational Level No formal education Primary Secondary Tertiary	0.57 (0.22, 1.48) 0.80 (0.48, 1.35) 1.02 (0.80, 1.31) Ref	0.248 0.407 0.850	1.24 (0.45, 3.42) 0.87 (0.51, 1.49) 0.96 (0.72, 1.28) Ref	0.681 0.602 0.796
Working Status Not working Working	1.23 (0.97, 1.57) Ref	0.090	1.34 (1.01, 1.77) Ref	0.003
Household Income* B40 M40 T20	1.02 (0.65, 1.59) 1.13 (0.69, 1.86) Ref	0.928 0.619		

* Bottom 40% (B40) representing low income, Middle 40% (M40) representing middle income, and Top 20% representing high income (T20).

## Discussion

This study highlights important factors associated with gestational diabetes mellitus (GDM) among women with advanced maternal age in Malaysia, providing valuable insights into demographic and socioeconomic disparities. The prevalence of GDM in this study was 33.7%, which is substantially higher than the pooled prevalence of GDM in Asia, reported at 11.5% [18,19], and the global prevalence of 10.9% based on a meta-analysis of 3258 studies [20]. The higher prevalence observed may be attributed to differences in diagnostic criteria, screening methods, and study settings, as well as the rising prevalence of obesity in the population [21]. These findings align with data from China, where GDM prevalence among women of advanced maternal age was recorded at 37.1%, with an 8% increase in GDM risk for every additional year of maternal age [22]. This underscores the critical need for heightened attention to GDM risk in older maternal populations.

Ethnicity emerged as a significant determinant of GDM, with Indian women demonstrating the highest prevalence and significantly greater odds of developing GDM. Similar patterns were observed for Malay, Chinese, and Other Bumiputera women, compared to other ethnicities. This finding aligns with previous studies suggesting ethnic disparities in metabolic and gestational outcomes, possibly influenced by genetic predispositions, dietary patterns, and lifestyle factors [23–27]. A study in Malaysia found that the ‘Cereals-confectionaries’ dietary pattern was significantly associated with abnormal glucose tolerance and this pattern was characterized by high-glycaemic index foods such as white rice, white bread, brown rice, flavoured rice, and traditional confectionaries, may contribute to insulin resistance and impaired glucose metabolism [28]. Furthermore, the higher prevalence of obesity among individuals of Indian ethnicity may further contribute to their elevated risk of GDM [29,30]. These results underscore the need for targeted interventions tailored to high-risk ethnic groups, including culturally sensitive dietary counselling and screening programs.

Working status was another significant factor in the multivariate analysis, with non-working women having 34% higher odds of developing GDM than working women. This contrasts with the univariate analysis, where working status was not significantly associated with GDM (p = 0.090). This discrepancy is explained by confounding due to ethnicity. Further analysis revealed that while both the Chi-square test (p = 0.090) and a single-variable logistic regression (p = 0.090) showed no significant association between working status and GDM, the association became significant (p = 0.029) when ethnicity was included in the logistic regression model. These results suggest that ethnicity functions as a suppressor variable, masking the relationship between working status and GDM in the absence of adjustment. This highlights the importance of multivariate analysis in revealing independent effects that may be obscured in univariate analyses. Consequently, this association may reflect differences in physical activity levels, socioeconomic stressors, and access to healthcare services [31]. Addressing these disparities through community-based health promotion programs and improving healthcare accessibility for non-working women could help mitigate this risk.

The significantly higher prevalence of GDM among women with advanced maternal age underscores the importance of enhanced preconception and antenatal counselling [31–33]. Healthcare providers should use this information to guide women in making informed decisions about the timing of childbearing. This is especially relevant given the increasing trend of delayed parenthood and its associated risks. Early screening, lifestyle interventions, and tailored education programs should be prioritized to address the rising burden of GDM, particularly among high-risk groups.

Furthermore, community-based non-pharmacological interventions for managing GDM offer effective and accessible alternatives to pharmacological treatments [34]. Evidence suggests that self-management programs significantly improve self-efficacy, lifestyle behaviours, and postprandial blood glucose levels, making them highly beneficial for GDM patients. Medical nutrition or diet therapy has also shown notable efficacy in lowering postprandial blood glucose compared to routine care, while combined diet and exercise interventions further reduce maternal weight gain more effectively than diet alone. Interestingly, digital health interventions, such as smartphone apps and telehealth, have shown to improve glycaemic control in pregnant women with GDM, reducing the need for caesarean delivery and the risk of foetal macrosomia [35]. There is currently a smartphone app being developed as part of the Explore-MYGODDESS study to evaluate the acceptability and feasibility of a culturally tailored digital diabetes prevention intervention for women with gestational diabetes mellitus in Malaysia, aiming to support dietary and lifestyle modifications, as well as addressing barriers to effective diabetes prevention [36,37]. However, more robust evidence is needed before these interventions can be recommended as a replacement for regular clinic follow-ups.

This study has several notable strengths. First, it utilizes nationally representative data from the NHMS 2022, ensuring the findings are generalizable across Malaysia due to the survey’s robust sampling design. Second, the focus on women with advanced maternal age—a high-risk group for GDM—fills a critical gap in Malaysian literature, offering specific insights into this vulnerable population. Third, the identification of key sociodemographic factors, such as ethnicity and working status, provides actionable insights for targeted public health interventions. However, there are also limitations to consider. The cross-sectional design restricts the ability to establish causal relationships between GDM and sociodemographic factors. Additionally, reliance on self-reported data for variables such as employment status and socioeconomic factors introduces the possibility of recall bias or misreporting, though the use of validated questionnaires helps mitigate this concern. Lastly, the study does not explore behavioural factors (e.g., physical activity, diet) or genetic predispositions, which could provide deeper insights into GDM risk. Despite these limitations, the findings serve as a valuable foundation for future longitudinal and multi-dimensional research.

## Conclusion

This study underscores the high prevalence of gestational diabetes mellitus (GDM) among women with advanced maternal age in Malaysia, highlighting significant associations with sociodemographic factors such as ethnicity and working status. Indian women exhibited the highest risk, emphasizing the need for culturally tailored interventions targeting high-risk ethnic groups. The findings provide critical insights into the importance of early screening, lifestyle interventions, and community-based programs to mitigate the burden of GDM. Despite limitations such as the cross-sectional design and lack of behavioural or genetic data, this study contributes valuable evidence to guide public health policies and strategies such as implementing mandatory GDM screening for high-risk ethnic groups and promoting workplace health programs for non-working women to reduce GDM prevalence and its associated complications in Malaysia. Future research should assess whether reducing sedentary time in non-working women lowers GDM incidence and should also investigate behavioural determinants and genetic predispositions through longitudinal studies to enhance the understanding and management of GDM.

## References

[1] McIntyre HD, Catalano P, Zhang C, Desoye G, Mathiesen ER, Damm P. Gestational diabetes mellitus. Nat Rev Dis Primers. 2019;5(1):47. doi: 10.1038/s41572-019-0098-8 31296866

[2] Modzelewski R, Stefanowicz-Rutkowska MM, Matuszewski W, Bandurska-Stankiewicz EM. Gestational diabetes mellitus-recent literature review. J Clin Med. 2022;11(19):5736. doi: 10.3390/jcm11195736 36233604 PMC9572242

[3] Frick AP. Advanced maternal age and adverse pregnancy outcomes. Best Pract Res Clin Obstetrics Gynaecol. 2021;70:92–100. 10.1016/j.bpobgyn.2020.07.005 32741623

[4] Deng L, Ning B, Yang H. Association between gestational diabetes mellitus and adverse obstetric outcomes among women with advanced maternal age: a retrospective cohort study. Medicine (Baltimore). 2022;101(40):e30588. doi: 10.1097/MD.0000000000030588 36221369 PMC9542683

[5] Koo BK, Lee JH, Kim J, Jang EJ, Lee C-H. Prevalence of gestational diabetes mellitus in Korea: a national health insurance database study. PLoS One. 2016;11(4):e0153107. doi: 10.1371/journal.pone.0153107 27046149 PMC4821493

[6] Bashir M, E Abdel-Rahman M, Aboulfotouh M, Eltaher F, Omar K, Babarinsa I, et al. Prevalence of newly detected diabetes in pregnancy in Qatar, using universal screening. PLoS One. 2018;13(8):e0201247. doi: 10.1371/journal.pone.0201247 30074993 PMC6075760

[7] Shivashri C, Hannah W, Deepa M, Ghebremichael-Weldeselassie Y, Anjana RM, Uma R, et al. Prevalence of prediabetes and type 2 diabetes mellitus in south and southeast Asian women with history of gestational diabetes mellitus: systematic review and meta-analysis. PLoS One. 2022;17(12):e0278919. doi: 10.1371/journal.pone.0278919 36508451 PMC9744276

[8] Sweeting A, Wong J, Murphy HR, Ross GP. A clinical update on gestational diabetes mellitus. Endocr Rev. 2022;43(5):763–93. doi: 10.1210/endrev/bnac003 35041752 PMC9512153

[9] Altemani AH, Alzaheb RA. The prevention of gestational diabetes mellitus (The role of lifestyle): a meta-analysis. Diabetol Metab Syndr. 2022;14(1):83. doi: 10.1186/s13098-022-00854-5 35706048 PMC9199329

[10] Gajera D, Trivedi V, Thaker P, Rathod M, Dharamsi A. Detailed review on gestational diabetes mellitus with emphasis on pathophysiology, epidemiology, related risk factors, and its subsequent conversion to type 2 diabetes mellitus. Hormone Metabolic Res. 2023;55(5):295–303. 10.1055/a-2061-9441 36963428

[11] Health IP. Technical report national health & morbidity survey: maternal and child health. Ministry of Health Malaysia; 2023.

[12] Health IfP. National Health and Morbidity Survey (NHMS) 2016: maternal and child health. Vol. II: Findings. Ministry of Health, Malaysia; 2016.

[13] Management of diabetes in pregnancy Malaysia. Ministry of Health Malaysia. 2017. https://www.moh.gov.my/moh/resources/Penerbitan/CPG/Endocrine/1a.pdf

[14] IPH. Technical Report National Health & Morbidity Survey: Maternal and Child Health. Malaysia; 2023.

[15] MOH. Clinical practice guidelines: management of diabetes in pregnancy. Malaysia; 2017.

[16] International Association of Diabetes and Pregnancy Study Groups Consensus Panel, Metzger BE, Gabbe SG, Persson B, Buchanan TA, Catalano PA, et al. International association of diabetes and pregnancy study groups recommendations on the diagnosis and classification of hyperglycemia in pregnancy. Diabetes Care. 2010;33(3):676–82. doi: 10.2337/dc09-1848 20190296 PMC2827530

[17] Padzil MSAM. Malaysian household poverty determinant by using model selection & model averaging. 2023.

[18] Lee KW, Ching SM, Ramachandran V, Yee A, Hoo FK, Chia YC, et al. Prevalence and risk factors of gestational diabetes mellitus in Asia: a systematic review and meta-analysis. BMC Pregnancy Childbirth. 2018;18(1):494. doi: 10.1186/s12884-018-2131-4 30547769 PMC6295048

[19] Paulo MS, Abdo NM, Bettencourt-Silva R, Al-Rifai RH. Gestational diabetes mellitus in Europe: a systematic review and meta-analysis of prevalence studies. Front Endocrinol (Lausanne). 2021;12:691033. doi: 10.3389/fendo.2021.691033 34956073 PMC8698118

[20] Zhang F, Dong L, Zhang C, Li B, Wen J, Gao W. Increasing prevalence of gestational diabetes mellitus in Chinese women from 1999 to 2008. Diabetic Med. 2011;28(6):652–7. 21569085 10.1111/j.1464-5491.2010.03205.x

[21] Nunes JS, Ladeiras R, Machado L, Coelho D, Duarte C, Furtado JM. The influence of preeclampsia, advanced maternal age and maternal obesity in neonatal outcomes among women with gestational diabetes. Revista Brasileira de Ginecologia e Obstetrícia. 2020;42(10):607–13. 32559795 10.1055/s-0040-1710300

[22] Han Y, Tong M, Jin L, Yu J, Meng W, Ren A. Maternal age at pregnancy and risk for gestational diabetes mellitus among Chinese women with singleton pregnancies. Int J Diabetes Dev Countries. 2021;41:114–20.

[23] Pagel KA, Chu H, Ramola R, Guerrero RF, Chung JH, Parry S, et al. Association of genetic predisposition and physical activity with risk of gestational diabetes in nulliparous women. JAMA Netw Open. 2022;5(8):e2229158. doi: 10.1001/jamanetworkopen.2022.29158 36040739 PMC9428742

[24] Hassani Zadeh S, Boffetta P, Hosseinzadeh M. Dietary patterns and risk of gestational diabetes mellitus: a systematic review and meta-analysis of cohort studies. Clin Nutr ESPEN. 2020;36:1–9. doi: 10.1016/j.clnesp.2020.02.009 32220350

[25] Jääskeläinen T, Klemetti MM. Genetic risk factors and gene-lifestyle interactions in gestational diabetes. Nutrients. 2022;14(22):4799. doi: 10.3390/nu14224799 36432486 PMC9694797

[26] Yong HY, Mohd Shariff Z, Mohd Yusof B-N, Rejali Z, Appannah G, Bindels J, et al. The association between dietary patterns before and in early pregnancy and the risk of gestational diabetes mellitus (GDM): data from the Malaysian SECOST cohort. PLoS One. 2020;15(1):e0227246. doi: 10.1371/journal.pone.0227246 31923230 PMC6953856

[27] Li L-J, Huang L, Tobias DK, Zhang C. Gestational diabetes mellitus among Asians - A systematic review from a population health perspective. Front Endocrinol (Lausanne). 2022;13:840331. doi: 10.3389/fendo.2022.840331 35784581 PMC9245567

[28] Hasbullah FY, Mohd Yusof B-N, Shyam S, Abdul Ghani R, Mohamed Khir HI. Dietary patterns associated with abnormal glucose tolerance following gestational diabetes mellitus: the MyNutritype study. Nutrients. 2023;15(12):2819. doi: 10.3390/nu15122819 37375723 PMC10302866

[29] Chong CT, Lai WK, Mohd Sallehuddin S, Ganapathy SS. Prevalence of overweight and its associated factors among Malaysian adults: findings from a nationally representative survey. PLoS One. 2023;18(8):e0283270. doi: 10.1371/journal.pone.0283270 37531379 PMC10395944

[30] Weir TL, Majumder M, Glastras SJ. A systematic review of the effects of maternal obesity on neonatal outcomes in women with gestational diabetes. Obes Rev. 2024;25(7):e13747. doi: 10.1111/obr.13747 38679418

[31] Hill-Briggs F, Adler NE, Berkowitz SA, Chin MH, Gary-Webb TL, Navas-Acien A. Social determinants of health and diabetes: a scientific review. Diabetes Care. 2020;44(1):258. 33139407 10.2337/dci20-0053 PMC7783927

[32] Correa-de-Araujo R, Yoon SSS. Clinical outcomes in high-risk pregnancies due to advanced maternal age. J Womens Health (Larchmt). 2021;30(2):160–7. doi: 10.1089/jwh.2020.8860 33185505 PMC8020515

[33] Glick I, Kadish E, Rottenstreich M. Management of pregnancy in women of advanced maternal age: improving outcomes for mother and baby. Int J Womens Health. 2021;13:751–9. doi: 10.2147/IJWH.S283216 34408501 PMC8364335

[34] Igwesi-Chidobe CN, Okechi PC, Emmanuel GN, Ozumba BC. Community-based non-pharmacological interventions for pregnant women with gestational diabetes mellitus: a systematic review. BMC Womens Health. 2022;22(1):482. doi: 10.1186/s12905-022-02038-9 36447189 PMC9710028

[35] Leblalta B, Kebaili H, Sim R, Lee SWH. Digital health interventions for gestational diabetes mellitus: a systematic review and meta-analysis of randomised controlled trials. PLOS Digit Health. 2022;1(2):e0000015. doi: 10.1371/journal.pdig.0000015 36812531 PMC9931335

[36] Sobri NHM, Ismail IZ, Hassan F, Papachristou Nadal I, Forbes A, Ching SM, et al. Protocol for a qualitative study exploring the perception of need, importance and acceptability of a digital diabetes prevention intervention for women with gestational diabetes mellitus during and after pregnancy in Malaysia (Explore-MYGODDESS). BMJ Open. 2021;11(8):e044878. doi: 10.1136/bmjopen-2020-044878 34446477 PMC8395347

[37] Benton M, Iman I, Goldsmith K, Forbes A, Ching SM, Papachristou Nadal I, et al. A mobile phone app for the prevention of type 2 diabetes in Malaysian women with gestational diabetes mellitus: protocol for a feasibility randomized controlled trial. JMIR Res Protoc. 2022;11(9):e37288. doi: 10.2196/37288 36074545 PMC9501684

